# Perspectives on Microbiome Therapeutics in Infectious Diseases: A Comprehensive Approach Beyond Immunology and Microbiology

**DOI:** 10.3390/cells13232003

**Published:** 2024-12-04

**Authors:** Hoonhee Seo, Sukyung Kim, Samuel Beck, Ho-Yeon Song

**Affiliations:** 1 Human Microbiome Medical Research Center (HM·MRC), School of Medicine, Soonchunhyang University, 22, Soonchunhyang-ro, Sinchang-myeon, Asan-si 31538, Chungnam-do, Republic of Korea; 2 Center for Aging Research, Department of Dermatology, Chobanian & Avedisian School of Medicine, Boston University, J-607, 609 Albany, Boston, MA 02118, USA; 3 Department of Microbiology and Immunology, School of Medicine, Soonchunhyang University, 31, Suncheonhyang 6-gil, Cheonan-si 31151, Chungnam-do, Republic of Korea

**Keywords:** microbiome therapeutics, infectious disease, resistance defense, tolerance defense, cooperative defense system

## Abstract

Although global life expectancy has increased over the past 20 years due to advancements in managing infectious diseases, one-fifth of people still die from infections. In response to this ongoing threat, significant efforts are underway to develop vaccines and antimicrobial agents. However, pathogens evolve resistance mechanisms, complicating their control. The COVID-19 pandemic has underscored the limitations of focusing solely on the pathogen-killing strategies of immunology and microbiology to address complex, multisystemic infectious diseases. This highlights the urgent need for practical advancements, such as microbiome therapeutics, that address these limitations while complementing traditional approaches. Our review emphasizes key outcomes in the field, including evidence of probiotics reducing disease severity and insights into host-microbiome crosstalk that have informed novel therapeutic strategies. These findings underscore the potential of microbiome-based interventions to promote physiological function alongside existing strategies aimed at enhancing host immune responses and pathogen destruction. This narrative review explores microbiome therapeutics as next-generation treatments for infectious diseases, focusing on the application of probiotics and their role in host-microbiome interactions. While offering a novel perspective grounded in a cooperative defense system, this review also addresses the practical challenges and limitations in translating these advancements into clinical settings.

## 1. Introduction

According to the World Health Organization’s (WHO) World Health Statistics 2023, global population health has significantly improved since the 2000s [1]. Notably, over the past two decades, life expectancy increased from 67 to 73 years, aligning with the decline in the incidence and mortality of infectious diseases [1]. However, 1 in 5 deaths is still caused by infections, and six out of the top 10 causes of death in low-income countries are infectious diseases [2]. Meanwhile, global health indicators saw a setback during the COVID-19 pandemic, causing approximately 15 million excess deaths in 2020 and 2021 [3]. This serves as a stark reminder that the emergence and re-emergence of infectious diseases remain significant risks [4]. WHO Director-General Dr. Tedros Adhanom Ghebreyesus emphasizes the importance of learning from the past two decades, including the tragedies of the pandemic era [1].

Although medicinal folklore has been used to treat infections since ancient cultures predating BC, humanity struggled with infections and epidemics until the 17th century [5]. During this time, ‘little beasties’ were observed under a microscope, abiogenesis was refuted, and the question of “What is the nature of infectious diseases?” was addressed [6,7]. The development of vaccines in the late 18th century and the advent of antibiotics, antivirals, and antifungals in the 20th century marked significant progress in combating infectious diseases [8,9,10,11]. However, pathogens quickly developed resistance mechanisms, contributing to a decline in the discovery of new drugs and treatments [12,13]. The emergence of novel pathogens and the continuous evolution of existing ones continue to challenge humanity [14]. Similarly, vaccine development faces difficulties such as rapidly evolving pathogens, high sequence variability, and complex viral antigens [15]. Thus, while vaccines and antimicrobials are crucial, they provide a valuable but incomplete perspective from the fields of immunology and microbiology [16].

In infectious disease outbreaks, clinical solutions traditionally focus on efficient pathogen destruction [17]. However, the COVID-19 pandemic emphasized the complexity of infectious diseases as multisystemic conditions, indicating that this perspective needs to be revised to understand survival from infectious diseases [16,17]. This highlights the need to understand how we survive infections, which may differ from standard approaches to treating infectious diseases [16]. For instance, antivirals may be effective in patients with “mild” COVID-19 by shortening the infection duration and reducing transmission. However, in severe cases requiring hospitalization and intensive care, antiviral-based strategies may not align with the needs of medical staff and patients fighting for their lives [18]. Effective response to an infectious disease outbreak necessitates a multifaceted and holistic approach [16,17].

There has always been a gap between treating infectious diseases and understanding the mechanisms promoting survival [19]. Moving beyond the traditional view of infectious diseases is imperative to enhance the treatment [17]. In addition to the resistance strategies of the host immune response and pathogen destruction, there is a need to develop disease-tolerance drugs that alleviate pathologies or promote physiological functions despite the presence of infection [16,17]. Against this backdrop, this narrative review aims to explicitly address the two mechanisms of resistance and tolerance in treating infectious diseases, particularly through the lens of the microbiome, which has recently gained attention as a next-generation treatment. This review also proposes developmental directions based on the convergence of these approaches.

## 2. Human Microbiome Therapeutics

“Microbiome” is a term derived from the combination of “micro” and “biome”, referring to the entire habitat of microorganisms, their genomes, and surrounding environmental conditions [20,21]. According to the Human Microbiome Project (HMP) Consortium report, human feces contain 4180 bacterial species, while the buccal mucosa, anterior nares, supragingival plaque, and posterior fornix harbor 775, 857, 1267, and 255 species, respectively [22]. In total, a “reference man” carries about 38 trillion bacteria, roughly equal to the 30 trillion human cells [23]. This newfound understanding of scale underscores the significance of the human microbiome. MetaHIT (The European Union Project on Metagenomics of the Human Intestinal Tract) characterized the genetic potential, revealing 3.3 million microbial genes in human feces, approximately 150 times more than the human genome’s 23,000 genes [24]. The shared pool contains around 536,000 unique genes: 99.1% are bacterial, with the remainder primarily from archaea and only 0.1% from eukaryotic and viral sources [24].

These microbial communities profoundly impact host physiology, influencing energy metabolism—converting nutrients into energy—and contributing to inflammation, bile acid metabolism, and body weight regulation through interactions with diet, genetics, and gut motility [25]. Consequently, the human microbiome is referred to as our second genome or other genome [26,27]. It is implicated in various diseases, including infectious diseases, inflammatory bowel disease, multiple sclerosis, diabetes, allergies, asthma, autism, and cancer [28]. However, these associations are primarily based on correlational studies, highlighting the need for further research to validate therapeutic potential.

This recognition led to Human Microbiome Therapeutics being named one of the World Economic Forum’s Top 10 Emerging Technologies in 2014 [29], catalyzing the rapid development of microbiome-targeting treatments [30]. The U.S. Food and Drug Administration (FDA) issued guidelines for live biotherapeutic products [31]. In 2022, REBYOTA, a microbiome treatment for recurrent *Clostridium difficile* infection, became the first FDA-approved product [32]. In 2023, Vowst, the inaugural oral microbiota-based product, also gained FDA approval specifically for recurrent *C. difficile* infection [33]. Recent advances include identifying biomarkers like *Akkermansia muciniphila*, which is linked to metabolic health, and targeting gut microbial pathways involved in antibiotic resistance and infections [34,35].

## 3. Probiotics: An Ancestral Approach to Modern Microbiome Therapeutics and Their Role in Treating Infectious Diseases

The term “probiotic” stems from the Latin “pro” (for, in favor of) and Greek “bios” (life) and is defined as “Live microorganisms which, when administered in adequate amounts, confer a health benefit on the host” [36,37]. Probiotic applications, not confined to a specific definition, encompass single or multiple strains and are utilized in both live and dead forms, sometimes in conjunction with immune stimulants like prebiotics and synbiotics [38]. The roots of probiotics trace back to the dawn of human history, closely intertwined with the advent of agriculture around 10,000 years ago. The journey from folk medicine to modern medicine began with the pioneering work of Russian scientist Elie Metchnikoff in the early 1900s [39]. Consequently, probiotics exhibit various effects, from providing nutritional benefits to impacting various bodily systems, including the intestines, brain, and skin. They are extensively researched in conditions such as irritable bowel disease, allergies, diabetes, cancer, and infectious diseases [40].

Probiotics exert their effects through diverse mechanisms, including modifying intestinal pH, producing antimicrobial compounds, competing with pathogens for binding sites and nutrients, and stimulating immune cells [41]. Consequently, probiotics have been a focal point in addressing infectious diseases such as antibiotic-associated diarrhea, pediatric diarrhea, and travelers’ diarrhea. Notably, a report states that two-thirds of randomized controlled trials on probiotics focus on infectious diseases [42]. Given the close relationship between the activity of probiotic bacteria and the host’s gastrointestinal condition, coupled with changes in the intestinal microbial population, they hold significant potential as alternatives to antibiotics for preventive and therapeutic purposes in gastrointestinal infectious diseases [43].

Nevertheless, an in-depth understanding of the molecular capabilities of probiotic bacteria is expanding the scope beyond gut-microbe interactions. The subsequent paragraphs briefly outline the mechanisms by which probiotics inhibit pathogenic microorganisms.

## 4. Mechanism of Action of Probiotics in Inhibiting Pathogenic Microorganisms

Antimicrobial compounds produced by probiotic bacteria directly inhibit competing enteropathogens in the gastrointestinal tract, preventing pathogenic colonization [43]. Bacteriocins such as nisin, plantaricin, and lacticin (2–10 kDa) play a crucial role by attaching to microbial cells and penetrating phospholipid membranes [44,45]. In Gram-positive bacteria, they primarily inhibit peptidoglycan synthesis, forming pores. In contrast, in Gram-negative bacteria, DNA, RNA, and protein metabolism inhibition occurs by targeting RNA polymerase, DNA gyrase, and aspartyl-tRNA synthetase [46]. For example, *Lactobacillus rhamnosus* produces bacteriocins effective against Clostridium difficile, which causes severe gastrointestinal infections [47,48]. Bacteriocins also depolarize target cell membranes, dissolve cell walls, and exhibit enzymatic activities like DNase, RNase, and phospholipase [46]. Additionally, uncharacterized bacteriocin-like inhibitory substances with similar activity are presented [49]. Probiotics engage in carbohydrate fermentation, producing organic acids like acetic acid, formic acid, succinic acid, and lactic acid [50]. These acids diffuse into microbial cells and acidify the cytoplasm, disrupting glycolysis, signal transduction, and metabolic pathways while increasing osmolarity and turgor pressure [50,51,52]. Other critical antimicrobial mechanisms involve hydrogen peroxide, causing highly deleterious DNA damage [53], siderophores, iron-chelating ligands sequestering essential iron [54], and biosurfactants, amphipathic molecules inducing permeability through detergent-like effects, causing leakage and dissolution of cell membranes [55,56].

Probiotics exhibit adhesion properties that contribute to a competitive exclusion effect, preventing intestinal pathogens from attaching to intestinal cells [57]. This adhesion relies on the physical interactions of van der Waals forces between surfaces [58]. Bacterial surface components, such as capsular polysaccharides, teichoic/lipoteichoic acids, and mucin-binding or collagen-binding proteins, form multicellular aggregates [59]. This process involves clumping with pathogens and adhesion to epithelial cells through co-aggregation [60]. Mucin, extracellular matrix, or lectin-like proteins in the digestive tract further promote the colonization of probiotics [59].

Colonized probiotics enhance the integrity of the intestinal epithelial barrier, thereby preventing the invasion of harmful antigens [61]. Probiotics influence the apoptosis and proliferation of intestinal epithelial cells and elevate the levels of transmembrane proteins, occludin and claudin, and junction adhesion molecules at tight junctions, thereby strengthening the intestinal mechanical barrier [61]. They also stimulate goblet cells to increase mucin production and plasma cells to secrete active IgA [62,63,64]. Additionally, probiotics promote the secretion of cationic beta-defensin-2 from intestinal epithelial cells, permeabilizing the lipid bilayer membranes of pathogens and reinforcing both chemical and immune barriers [65]. Despite these promising mechanisms, translating these findings into standardized clinical applications remains a challenge.

Probiotics play a role in modulating the host’s innate and adaptive immune responses by regulating immune cells such as dendritic cells, macrophages, and B and T lymphocytes [66]. For instance, dendritic cells in Peyer’s patches present microbial antigens to naïve T cells in mesenteric lymph nodes (MLN), activating the immune response [67]. This process is initiated by the recognition of conserved molecular structures known as microbe-associated molecular patterns (MAMPs) by pattern recognition receptors (PRRs) [68]. Subsequently, the activation of naïve T cells occurs through coordinated interactions between antigen-presenting cells, which noncovalently bind infectious agent-derived antigenic peptides to the major histocompatibility complex (MHC) and the T cell receptor (TCR) along with CD4 or CD8 coreceptors [69]. *Lactobacillus* promotes Th1 differentiation and enhances macrophage phagocytic ability by secreting IFN-α1, IFN-β, TNF-α, and IL-12, which help prevent intestinal infections and certain foodborne diseases [70]. Additionally, *Enterococcus* has been shown to maintain intestinal immune homeostasis by reducing the expression of crucial Th2 immune genes such as IL4, IL5, and CCL26, attenuating the typical polarized helminth-mediated Th2 immune response [71]. Th17 cells eliminate fungal and extracellular bacterial infections that are not efficiently removed by Th1- and Th2-type immunity [72]. Interestingly, recent research indicates that segmented filamentous bacteria (SFB) induce the accumulation of Th17 cells in the intestines of various species, including mice [73]. Furthermore, probiotics can generate FoxP3 T cell responses in the small intestine and induce CD4 and CD8 T cell activation in the colon [74]. As illustrated by the provided examples, probiotic bacteria are known to alter the intestinal microbial community, increasing the activity of immune cells such as Th1, Th2, Th17, and Treg cells, as well as B cells [75]. However, variability in probiotic effects across individuals and a limited understanding of immune modulation for pathogen clearance in infectious diseases emphasizes the need for further research on microbiota-targeted therapies.

## 5. Expanding Understanding of Infectious Diseases in the Microbiome Era

Since 2005, the traditional Sanger-based approach to DNA sequence analysis using capillary sequencers has undergone a revolutionary transformation with the introduction of next-generation sequencing (NGS) technology, which has significantly increased sequencing data output [76,77]. Over the past two decades, the gradual evolution of culture-independent methods has empowered researchers to sequence microbial communities from environmental samples directly [78]. This shift has brought ecological awareness to microbiology, with metagenomics playing a pivotal role in understanding the influence of microbial communities on human health and disease [79]. Consequently, advancements in the study of the human gut microbiome have led to the discovery of novel functional genes, microbial pathways, antibiotic resistance genes, functional dysbiosis, and interactions and coevolution between the microbiota and the host [80]. For instance, metagenomics has been pivotal in identifying carbapenem-resistant *Enterobacteriaceae* (CRE) in clinical settings, helping to develop targeted infection control measures and guide therapeutic decisions [81].

In addition to genomics, new molecular biochemical analyses, such as transcriptomics, proteomics, and metabolomics, are now applied to evaluate microbial communities’ genome and gene products beyond the gastrointestinal tract. This includes ecosystems with low microbial densities, such as the skin, airway system, and urogenital tract [82]. These include ecosystems with low microbial densities, such as the skin, airway system, and urogenital tract [82]. However, current technologies face challenges, including sequencing errors, biases in data interpretation, and incomplete reference databases, which may hinder the accuracy and comprehensiveness of microbial profiling [83].

According to a report from the U.S. National Institutes of Health, microbiome research funding reached $466 million from 2012 to 2016, with infectious diseases being the largest category, followed by digestive diseases, neoplasms, respiratory diseases, genitourinary diseases, and endocrine/metabolic diseases [84]. In this microbiome era, the interaction between the gut microbiome and the host is being identified, broadening the understanding of microbiome therapeutics beyond the gastrointestinal tract to include areas such as the oral cavity and respiratory system [85,86].

## 6. Microbiome-Based Deciphering in Gastrointestinal Infectious Diseases

Through metagenomic analysis, imbalances in the intestinal microbiome associated with gastrointestinal infectious diseases have been identified. Infants suffering from acute and persistent diarrhea showed a collapse of indigenous anaerobic microbial communities such as *Bacillota* and *Bacteroides* and aberrant proliferation of facultative anaerobes *Pseudomonadota*. Major pathogenic lineages in diarrheal samples included *Klebsiella*, *Haemophilus*, *Rothia*, *Granulicatella*, *Chelonobacter*, and *Vibrio* [87]. Children with antibiotic-associated diarrhea experience long-term dysbiosis, and in this case, the disease is correlated with the abundance of *Bacteroides*, as opposed to *Lachnospiraceae* and amino acid biosynthesis pathways [88]. The β-diversity of the intestinal microbiome in subjects with travelers’ diarrhea significantly differed compared to healthy travelers. While α-diversity remains unchanged, a higher ratio of *Bacteroidota* to *Bacillota* characterizes the gut microbiome of those with diarrhea [89]. Metagenomic associations between *Helicobacter pylori* infection status and changes in the gastric microbial community were also identified. After settling in the stomach, *H. pylori* becomes the dominant species, leading to a decrease in the Shannon index and subsequent reduction in species diversity. Taxa of *Stenotrophomonas*, *Chryseobacterium*, *Pedobacter*, *Variovorax*, and *Pseudomonas*, along with pathways of dTDP-L-rhamnose biosynthesis and tetrapyrrole biosynthesis, were more abundant in infected subjects [90].

As our understanding of the gut microbiome deepens, it becomes increasingly clear that it is an essential participant in the classic interpretation of host-pathogen interactions [91]. Accordingly, many studies have shown that the intestinal microbiota affects pathogen infection in various ways, including direct bacterial antagonism, stimulation of host immunity, and bacterial metabolism [91]. Colonization resistance, which involves interactions between commensals and pathogens, is a key defense mechanism [92]. Through quorum sensing (QS), bacteria regulate behavior via secreted chemical signals, enhancing their competitive edge [93].

In line with Freter’s nutrient-niche hypothesis, whereby intestinal bacteria compete for niche space in intestinal niches such as dietary compounds, they simultaneously engage in substrate competition [94]. They release antimicrobial substances such as small short-chain fatty acids, hydrogen peroxide (H_2_O_2_), organic acids, bacteriocins, and lipopeptides, causing changes in environmental conditions [95]. Interactions between microbial components and pattern recognition receptors in the gut epithelium activate innate and adaptive immune responses, which are crucial for maintaining mucosal homeostasis. Immune cells, including dendritic cells, macrophages, T cells, and B cells, along with IgA secretion, generate effective responses without inducing excessive inflammation [96].

Gut microbial communities metabolize proteins and complex carbohydrates, synthesize vitamins, and mediate crosstalk between gut epithelial and immune cells, producing an immense array of metabolic by-products. Short-chain fatty acids (SCFAs), the most abundant microbial metabolites, activate G-protein coupled receptors (GPCRs), maintaining gut barrier integrity and preventing pathogen invasion [97]. A healthy and balanced composition of microbial communities, known as Eubiosis, contributes to metabolic functions, protects against pathogens, and provides nutrients and energy to the host. Dysbiosis, or microbial imbalance, can lead to infections such as *C. difficile* [98].

The role of the microbiome in health and infectious diseases has spurred the development of live biotherapeutic products (LBPs) [99]. In patients with *C. difficile* infection, 20 to 40% relapse even after standard treatment with metronidazole or vancomycin, as the indigenous microflora cannot recover after antibiotic use [100]. *C. difficile*-directed antibiotics are associated with a decrease in α-diversity and differential relative abundance of bacterial and fungal assemblages, potentially enhancing microbial dysbiosis, a crucial determinant of relapse [101]. Against this backdrop, fecal microbiota transplantation (FMT), which restores native intestinal microflora by introducing microorganisms from a healthy donor, has emerged and shown a 90% success rate for recurrent *C. difficile* infection [102]. In homeostasis after FMT, secondary bile acids, deoxycholic acid (DCA), and lithocholic acid (LCA), produced by 7α-dehydroxylation of primary bile acid deconjugation mediated by microorganisms such as *Clostridium scindens*, inhibit the spore and vegetative growth of *C. difficile* [103,104]. Additionally, signals and metabolites such as SCFAs from the microbiota stimulate the mucosa to strengthen epithelial tight junctions and form a thick mucus layer containing antimicrobial peptides and high concentrations of secreted immunoglobulins. They also provide signals such as IL-10, TGFβ, and IL-22 to maintain the mucosal immune system’s non-inflammatory tone and epithelial homeostasis [105]. In this trend, REBYOTA, a fecal microbiota product for rectal administration, is mentioned above, and Vowst, an oral-fecal microbiota [106]. Although concerns persist regarding the potential transmission of multidrug-resistant organisms FMT [107], it is also regarded as a vital approach for eradicating such carriers [108], driving the development of microbiome therapeutics from defined bacterial consortia to single bacterial strains [32]. To overcome limitations, fecal virome transplantation (FVT), which targets gut microbiota using bacteriophages, is emerging as a promising alternative [109,110].

## 7. Understanding Nonintestinal Infectious Diseases Based on Microbiome-Host Communication

Microbial communities forming a mutualistic relationship with mammalian hosts can influence various physiological functions by regulating the host’s immune system [111]. Studies show that specific bacteria in defined niches send distinct signals, influencing innate and adaptive immune systems. This, in turn, can lead to distal systemic outcomes from the colonization site [111]. Especially in respiratory infections, changes in the composition of the gut microbial community components due to diet, disease, or medical interventions (such as antibiotics) are closely related to alterations in immune responses and homeostasis in the airways through what is termed the ‘gut–lung axis’ [112].

This phenomenon involves bidirectional communication as part of the shared mucosal immune system [113]. Specifically, a well-developed gut microbiome may enhance resistance to pathogens causing respiratory infections. In contrast, alterations in the gut microbiome and its products during respiratory conditions may influence harmful pathogenic transformations [114]. This concept has been pivotal in microbiome research, notably in tuberculosis. Significant changes in intestinal microbial diversity have been observed in tuberculosis patients, characterized by a sharp decline in bacteria-generating SCFAs and related metabolic pathways [115]. SCFAs, potentially involved as chemical messengers in the gut-lung axis, regulate immune responses by binding to GPCRs, including GPR41, GPR43, and GPR109A. Depending on downstream signaling, these receptors can either promote inflammation or exert anti-inflammatory effects [116]. Therefore, the gut microbial community imbalance could precede and contribute to *Mycobacterium tuberculosis* infection. Concerningly, severe disruptions in the gut microbial community induced by anti-tuberculosis treatment may increase susceptibility to subsequent reinfection or relapse of tuberculosis [117]. In vivo experiments have shown that the bacterial imbalance in the gut microbial community following anti-tuberculosis treatment increased the tuberculosis bacilli burden. Interestingly, this increased susceptibility was reversed by the transplantation of untreated mice’s fecal microbial communities [118].

While commensal microbes are predominantly studied in the gut, they also exist on the mucosal surfaces throughout the body. The lungs are no exception, and although considered sterile for decades, recent years have increasingly clarified the presence of a true microbial community in the respiratory tracts of mammals [119]. The pulmonary microbial community is believed to modulate the risk and outcomes of COVID-19 by activating innate and adaptive immune responses. Consequently, an imbalance in the pulmonary microbial community may contribute to acute respiratory distress syndrome (ARDS) in COVID-19 [120]. Indeed, COVID-19 patients showed changes in the bacterial microbial community in the lower respiratory tract during virus infection. Patients exhibited a noticeably high abundance of opportunistic pathogens, especially *Acinetobacter baumannii* and *Candida* spp., which correlated positively with inflammatory markers [121]. In essence, SARS-CoV-2 infection, by disrupting lung microbiota eubiosis, may enhance the cytokine storm in the lungs, potentially leading to secondary pathogen infections in COVID-19 patients. Pathogen-associated molecular patterns (PAMPs) released from invading opportunistic pathogens are recognized by host innate lymphocytes, including macrophages and dendritic cells, through PRRs such as Toll-like receptors (TLRs), RIG-I-like receptors (RLRs), and NOD-like receptors (NLRs). This recognition induces the expression of proinflammatory factors through NF-κB signaling, interferons through IRF3 signaling, and numerous interferon-stimulated genes (ISGs) through JAK/STAT signaling [122]. These findings may aid in developing treatments for respiratory infections caused by SARS-CoV-2 [121].

Most Human Immunodeficiency Virus type 1 (HIV-1) infections occur through sexual transmission, including both vaginal–penile and anal intercourse. The local immune environment at the site of HIV exposure, influenced by distinct microbiota and tissue-specific factors, plays a crucial role in determining whether exposure during sex leads to productive infection [123]. The vaginal and penile immune milieus are shaped by local microbiomes, with anaerobic taxa like *Prevotella* increasing the local density of HIV target cells expressing CCR5 and CD4, thereby increasing the risk of HIV infection. Conversely, the presence of *Lactobacillus* in the vagina and *Corynebacterium* in the penile region is associated with reduced inflammation and lower infection risk [123]. Moreover, HIV-1 infection induces changes in diversity and composition, leading to disrupted epithelial barriers, pathogen translocation, increased local inflammation, and the activation of myeloid dendritic cells. This activation drives increased T cell activation and the loss of protective Th17 and Th22 cells, contributing to epithelial barrier breakdown and promoting microbial translocation, potentially fostering bacterial imbalance and inflammation [124]. The potential “crosstalk” between viral and bacterial pathogens in HIV-infected individuals could aid in understanding the role of microbial communities. This insight may facilitate the identification of new antiretroviral factors for use in novel therapeutics [125].

Microbiome research in infectious diseases, including highly resistant and notorious superbugs against antibiotics, is ongoing [126]. As an extension, developing next-generation probiotics is expected to reduce healthcare-associated infections [127]. However, research on the microbiome related to Biosafety level 4 (BSL4) viruses, such as Ebola, one of the deadliest pathogens, is nonexistent. Future studies are essential.

## 8. Microbiome Therapeutics in the Management of Infectious Disease

The manipulation of human microbial communities holds significant potential for mitigating the occurrence and severity of various human conditions and diseases. The biomedical research community is actively translating its understanding of microbial communities into beneficial medical therapies [128]. Microbial communities, which share an extensive interface with the host’s immune system, emerge as excellent candidates for biomarker development [129]. The Linear Discriminant Analysis Effect Size (LEfSe) is a valuable tool that facilitates the identification of genomic biomarkers characterized by statistical differences between biological groups [130].

Microbiome therapeutics aim to manipulate microbial communities through addition, subtraction, or control strategies to maintain homeostasis, resilience, and resistance to disturbances [131,132]. Rich taxonomic biomarkers in healthy individuals can be approached as potential targets, utilizing technologies such as new organism–media pairings based on culturomics [133,134]. Conversely, a targeted antimicrobial approach is feasible for diseases rich in biomarkers. This involves treating infections by targeting major pathogens while restoring beneficial and healthy microbial flora [135]. For instance, in a microbiome analysis study on pulmonary tuberculosis patients, the genus Bifidobacterium had the highest Linear Discriminant Analysis (LDA) score in healthy individuals, while Bacteroides dominated in patients [136]. This suggests the potential for an approach involving probiotics for the former and targeted antimicrobials for the latter. Moreover, the analysis of genetically engineered probiotics can be pursued to develop functional biomarkers [137]. However, engineered probiotics may carry risks such as unexpected microbiome dysbiosis or unintended immune reactions, which need to be thoroughly evaluated before clinical application [138].

Bidirectional communication between humans and their microbial cohabitants is evident, and this dialogue can significantly impact our health in various ways [139]. Bacterial extracellular vesicles (BEVs) from the microbial community of the host’s gut can enter the circulatory system, disseminating to distant organs and tissues. Consequently, interest has grown in therapeutic approaches related to BEVs involving interkingdom communication, nutrient delivery, and immune modulation [140]. BEVs contain numerous MAMPs/PAMPs, including LPS, lipoproteins, peptidoglycan, and bacterial nucleic acids. These engage immune and non-immune cells’ PRRs, potentially conferring protective immunity, immunological tolerance, or promoting host pathology [140]. BEVs are presumed to promote maturation and immunological tolerance, protecting against conditions like colitis or sepsis [141]. Simultaneously, the release of exosomes by viruses, parasites, fungi, and bacterial infections is gaining attention for its ability to stimulate or inhibit host immunity by providing antigens to antigen-presenting cells [142].

Comparative mouse studies between germ-free and colonized mice have demonstrated the significant impact of the gut microbial community on the metabolome in distant body sites, including the kidneys, liver, and plasma. These findings suggest that microbial communities influence the biochemical environment, impacting health and disease [143]. The interaction between microbes in the human body and their metabolites, influencing disease risk, is beginning to be elucidated. Discoveries in this field hold high potential for developing preventive and therapeutic strategies for complex diseases [143]. Strategies are being explored to decode the microbiome-metabolome dialog concerning commensals’ metabolic activity and immunoregulatory properties, particularly in conditions like respiratory fungal diseases. The goal is to develop personalized medical interventions for patients at high risk of infection [144]. The metabolism of dietary fiber by gut microbes influences allergic airway disease and hematopoiesis [145]. This metabolic activity, dependent on G protein-coupled receptor 41 (GPR41), generates bone marrow hematopoiesis characterized by enhanced phagocytic capacity in macrophages and dendritic cell precursors. This leads to lung seeding by impaired dendritic cells that promote T helper type 2 (TH2) cell effector function [145]. Additionally, indole-3-aldehyde, a tryptophan derivative produced by *Lactobacillus* in the gut microbiome, acts as an aryl hydrocarbon receptor ligand, contributing to the expression of IL-22 and regulating tolerance to *Candida albicans* in the vaginal microbiome [146]. Therapeutic approaches based on metabolites provide a direct and feasible strategy to address the impact of microbial imbalance on the host. Metabolite-based therapeutic strategies are becoming highly promising [147]. While these advances are promising, it is important to address regulatory hurdles, such as ensuring the safety and efficacy of live biotherapeutics and gaining approval for clinical applications, as well as ethical concerns surrounding interventions like FMT [148]. Microbiome therapeutics extend to various interventions, such as intragastric, intravaginal, and intranasal routes, for inhibiting infections in intestinal, urogenital/vaginal, and respiratory infections. Advancements in microbial community analysis tools and technologies are opening up new potential pharmaceutical drugs [149,150].

## 9. Complex Defense Strategies: Resistance and Tolerance in Microbiome Therapeutics

Traditionally, clinical solutions for infectious diseases have predominantly focused on efficiently eradicating pathogens. However, the COVID-19 pandemic has emphasized infectious diseases’ complexity and multisystemic nature, highlighting the need for a holistic approach to maximize survival [17]. In the context of COVID-19, the disease’s states and potential therapeutic goals vary. For mild initial infections (Phase 1), antiviral treatments like remdesivir aim to reduce symptom duration, minimize infectivity, and prevent progression to severe conditions [18]. In contrast, during Phase 3, characterized by systemic hyperinflammation, immunomodulatory agents, including corticosteroids, IL-6 inhibitors like tocilizumab, and IL-1 receptor antagonists like anakinra, are used to reduce systemic inflammation before causing multiorgan dysfunction [18]. Controversies arise, particularly in severe COVID-19 cases, as observations question the benefits of treatments like lopinavir–ritonavir and suggest delays in virus clearance with steroid use, potentially increasing the duration of hospital stays [151,152]. This highlights the gap between treating infections and understanding survival mechanisms.

Traditional immunology has focused on mechanisms directly attacking invading microbes to block or remove them. However, recognizing the importance of defense mechanisms limiting infection damage has gained prominence. Consequently, interpreting the host’s defense abilities is crucial from the perspectives of resistance and tolerance [19]. Hosts can evolve two types of defense mechanisms, resistance or tolerance, to enhance fitness when challenged by pathogens. Understanding these defenses allows for more efficient treatments for infectious diseases and better explanations of host-pathogen interactions [19]. The microbiome plays a role in evolving a complex mechanism called colonization resistance, including nutrient competition, competitive metabolic interactions, niche exclusion, and induction of host immune responses to inhibit pathogen growth [153]. This is associated with enhancing resistance. Probiotics, for instance, enhance non-immunological defenses, stimulate specific and nonspecific host immune responses, and serve as anti-infective defenses against antibiotic-related conditions like viral gastroenteritis and *C. difficile* infection [154]. A healthy gut microbiome, rich in *Bifidobacterium*, *Faecalibacterium*, *Ruminococcus*, and *Prevotella*, is associated with low systemic inflammation. Quantitative data from a randomized, quadruple-blinded, placebo-controlled trial in COVID-19 patients demonstrated that probiotics alleviated symptoms, increased SARS-CoV-2-specific IgM and IgG, and reduced nasopharyngeal viral load. This evidence highlights the potential of microbiome therapeutics in supporting host defenses [155]. Additionally, probiotics, regulating host immune responses, offer a complementary approach to managing the ‘cytokine storm’ during COVID-19 infection [156]. Case studies further support the model. For example, in COVID-19 patients, therapies enhancing gut microbiota diversity reduced pathogen colonization and regulated immune responses, leading to improved inflammatory control [157]. These observations underscore the integrated impact of microbiome-based interventions. When applied to a reaction norms model concerning disease severity based on infection intensities [158], the interpretation is that the curve of the reaction norm shifts upward due to enhanced resistance, simultaneously with an improvement in tolerance leading to a softened slope of the curve.

**Figure 1:**
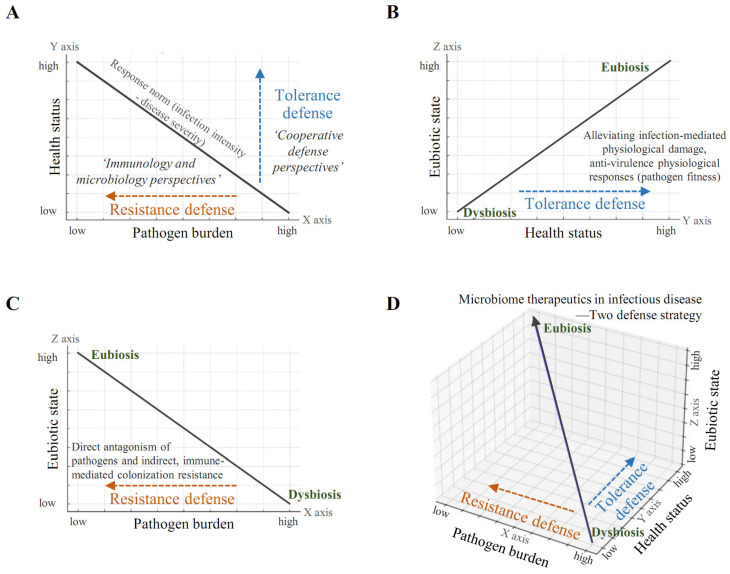
Establishing the Fundamentals of Infection-Microbiome Based on Resistance and Tolerance Defense in Microbiome-Integrated Cooperative Defense. By incorporating the microbiome perspective, this figure advances from the traditional immuno-microbiological view of resistance defense to a cooperative defense perspective that integrates tolerance defense, leading to a more comprehensive understanding. (**A**) The foundational concept of the infection-disease severity response model is that a higher pathogen burden correlates with increased disease severity, while a reduced burden corresponds to an improved health state. In this model, disease severity (Y-axis) inversely correlates with infection intensity (X-axis). In traditional immunology and microbiology, therapeutic strategies have largely focused on suppressing pathogen burden through antimicrobial agents and immune responses—An infection-resistant defense approach. However, in the case of infectious diseases, there is an increasing emphasis on tolerance defense, which, along with the perspective of cooperative defense, mitigates the physiological damage caused by infections. At this point, if tolerance defense is enhanced by microbiome therapeutics, the slope of the response norm graph flattens, indicating less severe physiological impacts despite pathogen presence. (**B**) The microbiome is closely linked to human health and disease. Within this context, a balanced microbiome (eubiosis) characterizes healthy individuals, indicating that a higher eubiotic state correlates with a healthier status. This connection aligns with the concept of tolerance defense in infectious diseases. (**C**) As the understanding of the microbiome expands, a fundamental approach to infectious diseases becomes possible. Dysbiosis, or microbial imbalance, is increasingly seen as a source of opportunistic pathogen proliferation and heightened pathogen burden. Here, an increased eubiotic state is associated with resistance defense. Microbiome therapeutics influence the improvement from dysbiosis to eubiosis while simultaneously leading to the enhancement of both resistance defense and tolerance defense. (**D**) In summary, the infection-disease severity response model, when connected to the eubiotic characteristics of the microbiome, provides a more fundamental understanding. Lower infection levels and better health are associated with a more eubiotic microbial state, while higher infection levels and poorer health correlate with dysbiosis. This interpretation suggests how these interconnected elements provide a novel perspective for the treatment of infectious diseases. Thus, microbiome therapeutics possess complex characteristics that encompass both traditional resistance and recent tolerance defense mechanisms, which can be understood through a cooperative defense system. This establishes a foundation for the “Microbiome-Integrated Infection-Disease Severity Response Model,” offering insights into the interactions among these variables and guiding the development of microbiome-based therapies.

As described above, Figure 1 illustrates the reaction norms model, reprocessed and adapted based on the data and insights from references [19,159], showcasing two defense strategies for microbiome therapeutics: resistance and tolerance, supported by quantitative data and case study outcomes.

## 10. Understanding the Mechanism of Microbiome Therapeutics Based on a Cooperative Defense System

For the past 50 years, research in immunology and infectious diseases has been rooted in assumptions based on activating immune responses and eliminating pathogens when infected. Efforts have been concentrated on developing vaccines and antibiotics and elucidating immune response mechanisms for infection control [160]. However, a challenge to this approach arose from cohort studies of SARS-CoV-2 infection, revealing similar virus burdens between symptomatic and asymptomatic patients [161]. The discovery of a cooperative defense system, allowing hosts to adapt to pathogens through disease resistance and antiviral defense health mechanisms, has initiated our understanding of this phenomenon [160]. Host-microbe interactions, rarely strictly defined as pathogenic, benign, or beneficial, have begun to be understood through cooperative metabolic strategies that can transition pathogens into a state of symbiosis [162]. As a result, hosts become healthy reservoirs for pathogens, promoting increased transmission to new hosts, such as asymptomatic persistent shedders. This can generate pathogen strains with higher fitness, as experimentally demonstrated [162]. The importance of maintaining a healthy microbiome in overcoming infectious diseases has been extensively documented, possibly interpreted as part of the detoxification of high-threat pathogens through cooperative metabolic strategies. A comprehensive interpretation from the perspective of the pathogen-microbiome-host immuno-metabolic network should follow. We need to learn more about host-encoded tolerance mechanisms based on the microbiome. Integrating the concept of tolerance into host-microbe studies for microbiome therapeutics is key to better understanding infectious disease treatments.

## 11. Fundamental to a Comprehensive Defense Perspective That Integrates the Microbiome with Conventional Immunology and Microbiology

Building upon the concepts discussed so far, this work establishes the fundamentals of infection-microbiome interactions based on resistance and tolerance defense mechanisms within an integrated microbiome-cooperative defense framework (Figure 1). By moving beyond the traditional immunology and microbiology perspectives of resistance defense to incorporate tolerance defense from a cooperative standpoint and further integrating the microbiome perspective, this work provides a more comprehensive and conceptual understanding. The foundational concept of the traditional infection-disease severity response model is that a higher pathogen burden correlates with increased disease severity, while a reduced burden is associated with improved health (Figure 1A). In this model, health status (Y-axis), which is linked to disease severity, inversely correlates with infection intensity (X-axis). Traditional therapeutic strategies in immunology and microbiology focus primarily on reducing pathogen burden through antimicrobials and immune responses—a resistance defense approach. However, an increasing emphasis on tolerance defense, which aims to mitigate physiological damage caused by infections from a cooperative defense perspective, provides a complementary layer to this model. The microbiome is closely connected to human health and disease. Within this context, a balanced microbial community (eubiosis) characterizes healthy individuals, indicating that a higher eubiotic state correlates with a healthier status (Figure 1B). This association suggests that tolerance defense mechanisms, such as mitigating excessive immune responses and physiological damage caused by infection, can be understood through the role of the microbiome. Moreover, a balanced microbiome may reduce pathogen virulence, supporting tolerance as a defense strategy by enhancing host fitness. As understanding of the microbial community expands, a fundamental approach to infectious disease management becomes possible. Dysbiosis, an imbalance in the microbiome, is increasingly recognized as a source of opportunistic pathogen proliferation and elevated pathogen burden (Figure 1C). An enhanced eubiotic state supports resistance defense by facilitating direct antagonism of pathogens through increased antimicrobial activity and immune strengthening and by contributing to colonization resistance, which reduces the pathogen burden. In summary, the infection-disease severity response model provides a more fundamental understanding when the eubiotic characteristics of the microbial community are represented along the Z-axis (Figure 1D). Lower infection levels and improved health are associated with a more eubiotic microbial state, whereas higher infection levels and poorer health potentially correlate with dysbiosis. This interpretation highlights how these interconnected elements suggest a new perspective for the treatment of infectious diseases. Consequently, microbiome therapeutics present a complex interplay of traditional resistance and emerging tolerance defense mechanisms that can be comprehensively understood within a cooperative defense system. This work lays the foundation for a “Microbiome-Integrated Infection-Disease Severity Response Model,” aiding in the fundamental understanding of the complex interactions between infectious diseases and microbial communities and guiding the development of microbiome-based therapies.

## 12. Challenges and Strategic Framework for Microbiome Therapeutics Development in Infectious Diseases

Despite the significant promise of microbiome therapeutics in managing infectious diseases, their application is accompanied by challenges, including potential intervention failures, risks, and inherent limitations, such as safety concerns [30]. For instance, variability in host responses, unintended microbial shifts, or insufficient long-term efficacy can lead to intervention failures. Safety issues, such as the risk of pathogen transmission, immune overactivation, or unforeseen side effects, highlight the need for stringent evaluation and regulatory oversight before widespread adoption. In addition to these practical concerns, the proposed model remains theoretical, with dynamic and non-linear interactions between host factors, microbiome composition, and pathogens requiring further empirical validation [159]. Furthermore, the roles of genetics, lifestyle, and environmental influences need deeper exploration to enhance therapeutic precision [163,164]. Studies on spousal and cohabitation dynamics provide valuable insights into how shared environments influence microbiome composition and function, but further research is required to establish their clinical relevance.

**Figure 2:**
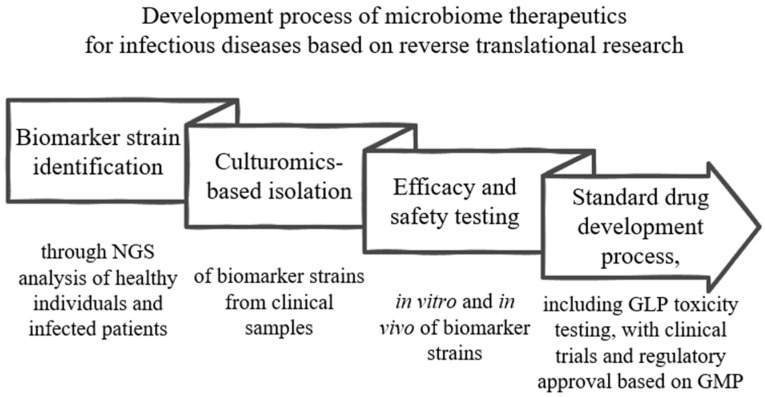
Development Process of Microbiome Therapeutics for Infectious Diseases Based on Reverse Translational Research. The figure illustrates a stepwise framework for the development of microbiome therapeutics targeting infectious diseases. It begins with the identification of biomarker strains through NGS analysis of healthy individuals and infected patients. The identified strains are then isolated using culturomics from clinical samples. This is followed by efficacy and safety testing, conducted in vitro and in vivo, to evaluate their therapeutic potential. The process concludes with the standard drug development pipeline, including Good Laboratory Practice (GLP) toxicity testing, as well as clinical trials and regulatory approval based on Good Manufacturing Practice (GMP) to ensure safety and efficacy. This framework highlights the systematic approach based on reverse translational research to develop microbiome-based treatments.

Given these challenges and opportunities, building on these considerations, we propose a systematic approach to the development of microbiome therapeutics for infectious diseases based on reverse translational research [165] (Figure 2). Next, the identified biomarker strains are isolated and cultivated from clinical samples using culturomics techniques [166]. These biomarker strains are then subjected to in vitro and in vivo efficacy testing to evaluate their potential therapeutic effects against the target infectious disease. The development progresses through the standard pharmaceutical pipeline to ensure safety and efficacy, including GMP toxicity testing, clinical trials, and regulatory approval [167]. This stepwise framework offers an effective therapeutic solution compared to conventional methods based on library screening.

## 13. Conclusions

The microbiome encompasses the composition of microbial communities, spatiotemporal heterogeneity/dynamics, network stability/resilience, and the co-evolutionary/holistic characteristics within the framework of the one health concept [168]. On the other hand, defense strategies against microbes are commonly perceived in terms of the immune system’s functionality in detecting and eliminating microbes by executing resistance mechanisms [21]. However, this perspective makes it difficult to understand infection response strategies based on host-microbe interactions from a genuine microbiome standpoint. Therefore, a shift in perspective is needed from “how to combat infectious diseases” to “how to survive infections” under the viewpoint of a cooperative defense system [16]. Microbiome therapeutics manage infections by restoring microbial balance and modulating immune responses, demonstrating potential in conditions like COVID-19 and *C. difficile*. Future studies should prioritize expanding microbiome-based applications to underexplored areas, such as antiviral therapies for respiratory infections and treatments for non-intestinal diseases. We conclude by introducing a new, comprehensive approach to infectious disease management, leveraging microbiome therapeutics to expand beyond traditional immunology and microbiology.

## Data Availability

Not applicable.
